# Ascorbyl palmitate-incorporated paclitaxel-loaded composite nanoparticles for synergistic anti-tumoral therapy

**DOI:** 10.1080/10717544.2017.1370619

**Published:** 2017-08-31

**Authors:** Min Zhou, Xin Li, Yuanyuan Li, Qiu’e Yao, Yue Ming, Ziwei Li, Laichun Lu, Sanjun Shi

**Affiliations:** a Department of Pharmacy, Institute of Surgery Research, Daping Hospital/The Third Affiliated Hospital, Third Military Medical University, Chongqing, China;; b Teaching Experimental Center, College of Pharmacy, Third Military Medical University, Chongqing, China

**Keywords:** Vitamin C, derivative, ascorbyl palmitate, paclitaxel, synergistic anticancer efficacy

## Abstract

A co-loaded drug delivery system based on ascorbyl palmitate that can transport various functional drugs to their targets within a tumor represents an attractive strategy for increasing the efficiency of anticancer treatment. In this study, we developed a dual drug delivery system to encapsulate ascorbyl palmitate (AP) and paclitaxel (PTX) for synergistic cancer therapy. AP, which is a vitamin C derivative, and PTX were incorporated into solid lipid nanoparticles (AP/PTX-SLNs), which were used to treat murine B16F10 melanoma that had metastasized to the lungs of mice. These nanoparticles were spherical with an average size of 223 nm as measured by transmission electron microscope and dynamic light scattering. *In vitro* cytotoxicity assays indicated that the AP/PTX-SLNs with an AP/PTX mass ratio of 2/1 provided the optimal synergistic anticancer efficacy. *In vivo*, AP/PTX-SLNs were revealed to be much more effective in suppressing tumor growth in B16F10-bearing mice and in eliminating cancer cells in the lungs than single drug (AP or PTX)-loaded SLNs via a synergistic effect through reducing the Bcl-2/Bax ratio. Furthermore, no marked side effects were observed during the treatment with the AP/PTX-SLNs, indicating that the co-delivery system with ascorbyl palmitate holds promising clinical potential in cancer therapy.

## 1. Introduction

Vitamin C (Vc), also known as ascorbate or l-ascorbic acid, is an important water-soluble vitamin in humans and is essential for carnitine, collagen and neurotransmitter biosynthesis. As a pharmacological agent, ascorbic acid has many applications, such as antioxidant, anti-atherogenic, and immunomodulator functions and the ability to prevent colds (Naidu, 2003b), etc. In the mid-twentieth century, a study hypothesized that cancer may be related to changes in connective tissue, which may be a consequence of vitamin C deficiency (Mc, 1959). In 1970 s, Ewan Cameron and Linus Pauling summarized a series studies on the antitumoral effects of high doses of vitamin C and concluded that ascorbic acid extended the lifespan of cancer patients and enhanced the quality of life (Cameron & Campbell, 1974; Cameron & Pauling, 1976). By contrast, several subsequent randomized controlled trials of high-dose oral vitamin C (10 g daily) failed to demonstrate similar benefits (Creagan et al., 1979; Moertel et al., 1985), and some even hold the idea that an overdose of vitamins may even increase the risk of cancer, opening the issue of therapeutic effectiveness to controversy. While recent studies showed that high dose of vitamin C exhibit anticancer effect only administered intravenously (approximately 40–400 mg/kg) or intraperitoneally (4 g/kg) (Padayatty et al., 2004; Chen et al., 2008; Verrax & Calderon, 2009; Takemura et al., 2010). Since then, numerous *in vitro* studies on both human and animal tumors have been carried out to elucidate the role of ascorbic acid in the prevention of different types of cancer, including studies of prostate (Pollard et al., 2010), pancreatic (Du et al., 2010, Espey et al., 2011), hepatocellular (Lin & Chuang, 2010), ovarian (Chen et al., 2008), colon (Ha et al., 2009; Pathi et al., 2011), mesothelioma (Takemura et al., 2010), neuroblastoma (Hardaway et al., 2012), and malignant melanoma (Kang et al., 2003; Miles et al., 2015) cell lines. Nevertheless, the *in vivo* application of ascorbate is currently limited by the very high blood concentrations that are required to achieve therapeutic levels in tumors (Ohno et al., 2009). In general, patients do not seem to well tolerate treatments of such high doses of the drug, and therefore, nanoparticle-based delivery is one approach that could be used to reduce the drug dose and improve drug pharmacokinetics. However, the formulation of drug delivery systems with ascorbic acid is rarely reported because this substance, which is highly soluble in water, is extremely sensitive to air, light, and heat and can be easily destroyed by prolonged storage or over processing (Naidu, 2003b). To overcome these problems, ascorbic acid can be chemically modified by esterification of the hydroxyl group with long-chain organic or inorganic acids. As a result, several more stable hydrophobized derivatives of ascorbate that retain the anti-cancer activity of ascorbate have been explored (Miwa et al., 1988; Lee et al., 2009; Sawant et al., 2011). Among them, ascorbyl palmitate has attracted extensive interest as an anticancer compound because of its lipophilic nature, allowing it to easily cross cell membranes (Banks & Kastin, 1985).

Ascorbyl palmitate (AP), a synthetic lipophilic derivative of ascorbic acid, has been used as a source of vitamin C and as an antioxidant for foods, pharmaceuticals, and cosmetics (Austria et al., 1997; Perrin & Meyer, 2003; Lee et al., 2009). Moreover, AP has been found to inhibit the cell proliferation and DNA synthesis of various cancer cells, including breast, colon, glioblastoma, skin, and brain cancer cells (Naidu, 2003a). Although AP is more stable than vitamin C, the therapeutic efficacy of AP is limited due to its water insolubility, rapid degradation (accelerated by metal ions and/or light), and low bioavailability. Therefore, new technologies including nanoparticles that can enhance its delivery efficacy and reduce the dose of administration for Vc while not reducing its anti-cancer efficacy are highly desired.

Solid lipid nanoparticles (SLNs) are novel nanocarriers with a size range of 10 to 1000 nm (Muller et al., 2000). They have attracted great attention as a good drug delivery system because of their unique structure and properties, such as excellent biocompatibility, controlled release of drug, good physicochemical stability, better accumulation in solid tumors through the enhanced permeability and retention (EPR) effect, and their ability to improve the bioavailability of drugs and to protect them from the external environment (Kristl et al., 2003; Maeda et al., 2013; Guney et al., 2014; Wang et al., 2015b; Qu et al., 2016; Guo et al., 2017). Recently, an increasing number of studies have reported nano-sized carriers designed for combination drug delivery to obtain enhanced and synergistic therapeutic efficacy in chemotherapy. Combination therapy is a promising strategy for anticancer treatment in clinical practice, which has the potential to not only reduce individual drug-related toxicity by reducing the dose of each agent required and achieve synergistic anticancer effects but also to suppress multi-drug resistance (MDR) by acting through different mechanisms (Wang et al., 2011; Wang et al., 2015c). More importantly, AP can act as both the lipid matrix which carries the drug and the chemotherapeutics. Recently, AP was used to modify the surface of liposomes to evaluate the potential of ascorbate as a novel ligand in the preparation of pharmaceutical nanocarriers with enhanced tumor-cell specific binding (D’Souza et al., 2008). However, the therapeutic effect and delivery of AP against cancer still remained uncertain.

In this study, to maximize the therapeutic efficacy of AP, we present an ascorbyl palmitate-based nanoparticle system in which both a derivative of Vc and chemotherapeutics paclitaxel (PTX) can be loaded for the treatment of cancer. Given its liposoluble nature, AP is an ideal candidate for loading into solid lipid nanoparticles. Our goal here was to evaluate the potential of AP-based nanoparticles combined with PTX (AP/PTX-SLNs) for killing cancer cells. We investigated the physiochemical properties and drug release behavior of this dual drug co-delivery system. The cellular uptake and synergistic anticancer effect of the AP/PTX-SLNs were also studied in the B16F10 cell line both *in vitro* and *in vivo*.

## 2. Materials and methods

### 2.1. Materials

Glyceryl monostearate (GMS) was purchased from Taiwei Pharmaceutical Co., Ltd (Shanghai, China). Dimethyldioctadecylammonium bromide (DDAB) and coumarin-6 were purchased from Sigma-Aldrich (St. Louis, MO). Ascorbyl palmitate (AP) was obtained from Tokyo chemical Industry Co., Ltd. Pluronic F-68 was supplied from Nanjing Well chemical Co., Ltd (Nanjing, Chain). Paclitaxel (PTX) was obtained from Haoxuanbio. Co., Ltd (Xi’an, China), and 3-(4,5-methylthiazol-2-yl)-2,5-diphenyltetrazolium bromide (MTT) was obtained from Amresco (Solon, OH). All other chemicals were reagent grade and used as received without further purification.

### 2.2. Cell culture and animals

The murine melanoma cell line B16F10 was obtained from the American Type Culture Collection (ATCC) and cultured with RPMI 1640 medium supplemented with 10% fetal bovine serum (FBS) and 1% penicillin/streptomycin. Female C57BL/6 mice (18–20 g) were supplied by the Laboratory Animal Center of the Third Military Medical University (Chongqing, China). All animal experiments were approved by the Institutional Animal Care and Ethic Committee of Third Military Medical University. The animals were maintained at animal care facility for at least one week with fresh diet and water before experiments.

### 2.3. Preparation and characterization of vitamin C derivative-based solid lipid nanoparticles for paclitaxel (AP/PTX-SLNs)

AP-incorporated composite SLNs loaded with paclitaxel were fabricated via a modified ultrasound method (Silva et al., 2011) under sterile condition. In brief, GMS (50 mg), DDAB (5 mg), PTX (2 mg) and AP (4 mg) were heated to 85 °C, which is 5–10 °C above the melting point of GMS. The aqueous phase containing Pluronic F-68 in double-distilled water (5 mL) was simultaneously heated to the same temperature. A pre-emulsion was obtained by adding the aqueous phase to the molten lipid phase. Then, an ultrasonic probe (6 mm diameter) was placed in this pre-emulsion via sonication in a VCX 130 Ultrasonic Cell Disrupter (SONICS). A power output with an amplitude of 50% was applied for 25 minutes at a temperature above the melting temperature, and AP/PTX-SLNs were formed after cooling the mixture to room temperature in an ice bath (Shi et al., 2012). AP-SLNs, PTX-SLNs and blank SLNs were prepared in the same way but without AP or/and PTX.

The particle size, polydispersity and zeta potential were measured by using a Nano ZS90 Zetasizer (Malvern, UK). In addition, the stability of AP-SLNs, PTX-SLNs and AP/PTX-SLNs were evaluated by measuring various physiochemical properties of SLNs, including the changes in the size and encapsulation efficiency after storing at 4 °C for one week. The morphology of the SLNs was examined using a transmission electron microscope (TEM) (TEM-1400 plus, JEOL). The entrapment efficiency (EE) of AP and PTX was calculated using high-performance liquid chromatography (HPLC). A reverse phase HPLC column (Kromasil ODS-1 C_18_, 5 μm, 150 × 4.6 mm) was used. The flow rate was set at 1 mL/min, and the temperature was set at 30 °C. To quantify AP, the mobile phase consisted of acetonitrile: 0.5% acetic acid at 95:5 v/v, and the wavelength of detection was 243 nm. For PTX, the mobile phase was composed of acetonitrile:water at 80:20 v/v, and the wavelength was 227 nm. The amounts of free drugs in the SLNs suspensions were separated via an ultrafiltration method using centrifugal filter tubes with a 10 kDa molecular weight cutoff (Millipore). The encapsulation efficiency was calculated by the following equation.

$$\mathrm{Encapsulation}\mathrm{efficiency}\%=[1-\frac{\mathrm{weight}\mathrm{of}\mathrm{free}\mathrm{drug}}{\mathrm{weight}\mathrm{of}\mathrm{the}\mathrm{feeding}\mathrm{drug}}]\times 100$$

$$\mathrm{Drug}\mathrm{loading}\mathrm{yield}\%=\frac{\mathrm{Weight}\mathrm{of}\mathrm{encapsulated}\mathrm{drug}\mathrm{in}\mathrm{SLNs}}{\mathrm{Total}\mathrm{weight}\mathrm{of}\mathrm{SLNs}}\times 100$$

The release profiles of AP and PTX from the SLNs were monitored according to a dialysis method (Wang et al., 2015a). Briefly, 1 mL of SLNs (containing 0.4 mg of PTX and 0.8 mg of AP) was placed in the dialysis bag (molecular weight cutoff of 10 kDa) and immersed into 100 mL of PBS (pH 7.4 or 5.5) containing 20% ethanol (Yoksan et al., 2010), then the entire system was incubated at 37 ± 1 °C under constant shaking at 100 rpm. At pre-determined time intervals, 1 mL of release media were removed from the dialysate and replaced by an equal volume of PBS/ethanol buffer. The amount of AP and PTX in the dialysate was determined by HPLC.

### 2.4. Differential scanning calorimetry (DSC)

The dried samples, including AP, PTX, blank SLNs, AP-SLNs, PTX-SLNs and AP/PTX-SLNs, were characterized using a DSC analyzer (DSC 200F3, NETZSCH). All samples were weighed and sealed in the aluminum cell and then heated from 25 °C to 350 °C at a rate of 10 °C/min under a nitrogen atmosphere.

### 2.5. *In vitro* cytotoxicity analysis

B16F10 cells and A549 cells were seeded at a density of 1 × 10^4^ cells per well in 96-well plates and grown to approximately 70% confluence prior to drug treatment. The culture media were replaced with 100 μL of fresh culture media. AP, PTX, AP + PTX (a mixture of AP and PTX), AP-SLNs, PTX-SLNs, AP/PTX-SLNs, or AP-SLNs + PTX-SLNs (a mixture of AP-SLNs and PTX-SLNs) were added to the cells. The concentrations of AP were 40, 20, 10, 5, 2.5 μM and the concentrations of PTX were 9.6, 4.8, 2.4, 1.2, 0.6 μM, respectively. Following incubation for 24 h, the cell viability was measured by 3-(4, 5-dimethythiazol-2-yl)-2, 5-diphenyl tetrazolium bromide (MTT) assays. The absorbance of each plate was read at 490 nm using a Spectra max plus microplate reader (Molecular Devices, CA). The concentrations of AP and PTX required to inhibit cell growth by 50% (IC_50_ value) were calculated. All the experiments were repeated five times.

To further study the synergy of AP/PTX-SLNs, the combination index (CI) was determined (Wang et al., 2015c). The CI analysis provides qualitative information regarding the nature of the drug synergism. The CI was calculated according to the following equation:

$$CI_{\mathrm{50}}=\frac{{\left(D\right)}_{A}}{{\left(D_{\mathrm{50}}\right)}_{A}}+\frac{{\left(D\right)}_{P}}{{\left(D_{\mathrm{50}}\right)}_{P}},$$

where (D_50_)_A_ and (D_50_)_P_ represent the IC_50_ values of AP alone and PTX alone, respectively. (D)_A_ and (D)_P_ represent the concentrations of AP and PTX, respectively, in the combination system at the IC_50_ value. A CI value <1 represents synergism, =1 represents addition, and >1 represents antagonism.

### 2.6. Cellular uptake studies

The cellular uptake and distribution of SLN formulations were characterized by fluorescence microscopy and flow cytometry. Briefly, B16F10 cells were seeded at a density of 2 × 10^5^ cells per well in 12-well culture plates and further cultured overnight. For imaging, coumarin-6 was incorporated into the SLNs to obtain the fluorescence-labeled composite nanoparticles. The coumarin-6 labeled formulation and free coumarin-6 at different concentrations were added to the cells without serum, and the cellular uptake was observed in a time- or dose-dependent manner. Following incubation at 37 °C, the B16F10 cells were washed twice with PBS, harvested, and analyzed using a Ti-S fluorescence microscope (Nikon, Tokyo, Japan) and a flow cytometer (Navios, Beckman, CA).

### 2.7. Cell apoptosis analysis

Cell apoptosis was assessed by fluorescence microscopy and flow cytometry (He et al., 2015). B16F10 cells were seeded at 5 × 10^5^ cells per well in 6-well plates and incubated overnight. The culture media were replaced with 1 mL of fresh culture media. AP solution, PTX solution, AP + PTX solution, AP-SLNs, PTX-SLNs, or AP/PTX-SLNs were added to the cells at a concentration of 2.05 μg/mL of AP or/and 1.0 μg/mL of PTX. After 24 h of incubation, the cells were rinsed with PBS, and the cell nuclei were then incubated with (4′-6-diamidino-2-phenylindole (DAPI) for 3–5 min. The morphology of the cell nuclei was analyzed using a Nikon fluorescence microscope. For flow cytometry, after culturing for 24 h, the floating and adherent cells were collected and stained with an Annexin V-FITC Apoptosis Detection Kit (Beyotime, China) according to the manufacturer’s instructions. The apoptotic cells were examined using a flow cytometer.

### 2.8. Western blot analysis of Bcl-2 and Bax

After 24-h treatment of AP, PTX, AP-SLNs, PTX-SLNs and AP/PTX-SLNs (AP: 4.05 μg/mL; PTX: 2.025 μg/mL), the media were aspirated, and the B16F10 cells were washed twice with ice-cold PBS. The proteins were harvested from the cells using cell lysis buffer (Beyotime, Shanghai, China) containing 1 mM PMSF (Beyotime, Shanghai, China). The protein concentrations were measured using a BCA Protein Assay Kit (Beyotime, Shanghai, China). The proteins were separated by 10% (for Bcl-2, Bax) SDS-polyacrylamide gels and electrophoretically transferred onto PVDF membranes (Millipore, MA). The membranes were blocked with 6% nonfat milk at 37 °C for 1 h and then incubated overnight with the corresponding primary antibodies (rabbit anti-Bax antibody (Abcam, Cambridge, UK), rabbit anti-Bcl-2 antibody (Abcam, Cambridge, UK), and β-actin antibody (Cell Signaling Technology, Danvers, MA)) at 4 °C. The blots were incubated with peroxidase-conjugated AffiniPure goat anti-rabbit or anti-mouse IgG (1:5000, ZSGB-BIO, Beijing, China) as the secondary antibodies for 1 h at 37 °C. The blots were detected with the Immobilon Western Chemiluminescent HRP Substrate (Millipore, MA). The protein levels were quantified via densitometry using LabQuant Software (Warwickshire, UK).

### 2.9. *In vivo* imaging assay

1,1-Dioctadecyl-3,3,3,3-tetramethylindodicarbocyanine (DiD, KeyGEN, China) was used to label the SLN formulations for *in vivo* imaging. In brief, 100 μg of DiD were added to the molten lipid phase followed by SLN preparation mentioned above. The mice were administered the SLN formulations and free DiD at an equivalent of 200 μg DiD per kg body weight. At the desired time interval, each group was anesthetized following injection with 3.5% chloral hydrate, and then the fluorescence signals of mice were recorded (Ex = 644 nm; Em = 667 nm) using an IVIS® Spectrum system (Caliper, Hopkington, MA) at pre-determined time points. Next, the mice were sacrificed, and the major organs from the euthanized animals were also imaged under the same set of parameters.

### 2.10. *In vivo* therapeutic experiments

An *in situ* murine lung tumor model was established by intravenous injection of 5 × 10^5^ B16F10 cells via the lateral tail vein into C57BL/6 mice (Chen et al., 2010). The mice were randomly divided into seven groups (AP, PTX, AP + PTX, AP-SLNs, PTX-SLNs, AP/PTX-SLNs and PBS). Beginning on day 9, the B16F10-bearing mice were administered i.v. injections of the various drug-containing formulations via the tail vein every day at doses of 4 mg PTX per kg body weight until day 16. The body weight was monitored every day. On day 21, the animals were sacrificed after six injections, and their B16F10-bearing lungs were collected, weighted and analyzed by H&E staining.

### 2.11. TUNEL assay

TUNEL assays were performed with a one-step TUNEL Apoptosis *in situ* detection kit (KeyGEN, Nanjing). Briefly, after treatment with various drug-loaded SLN formulations, both normal lungs and B16F10-bearing lungs were excised and fixed in 10% formaldehyde overnight, which were then paraffin-embedded and sectioned using a Leica microtome. The apoptotic cells in the tumor tissues were detected by a TUNEL assay. The dewaxed sections were stained green (Streptavidin-FITC), which revealed TUNEL-positive nuclei. Simultaneously, the cell nuclei were stained with DAPI. The samples were imaged using a fluorescence microscope.

### 2.12. Preliminary safety evaluation

The toxicity of the AP/PTX-SLNs was evaluated based on the health of the C57BL/6 mice (18–20 g). After i.v. injection of free AP, free PTX or the AP/PTX-SLNs (4 mg/kg of PTX), serum from each group was collected and assayed for the concentrations of proinflammatory cytokine interleukin-6 (IL-6) and anti-inflammatory cytokine interleukin-10 (IL-10) using commercial ELISA kits according to the manufacturer’s instructions (BOSTER, Wuhan, China). The absorbance at 450 nm was measured using a Spectra max plus microplate reader (Molecular Devices). Furthermore, major organs, including the heart, liver, spleen, lung, kidney and brain, were collected for histopathology investigation. Untreated mice were used as controls.

After treatment with various SLN formulations, the tissues were collected, fixed in 10% formaldehyde and then embedded in paraffin to prepare paraffin-embedded sections. The paraffin-embedded sections were stained with hematoxylin and eosin (H&E) for histopathologic evaluation. Injuries were examined microscopically for evidence of cellular damage and inflammation. All images of stained sections were captured using a light microscope (Olympus, Japan).

### 2.13. Statistics

All quantitative data were reported as the mean ± standard deviation from at least three separate experiments performed in triplicate, unless otherwise noted. Statistical significance was evaluated by Student’s *t*-test. For comparisons of more than two groups, one-way ANOVA followed by post hoc *t*-tests was performed and the levels of significance were set as *p* < .05.

## 3. Results and discussion

### 3.1. Preparation and characterization of AP/PTX-SLNs

**Figure 1.**
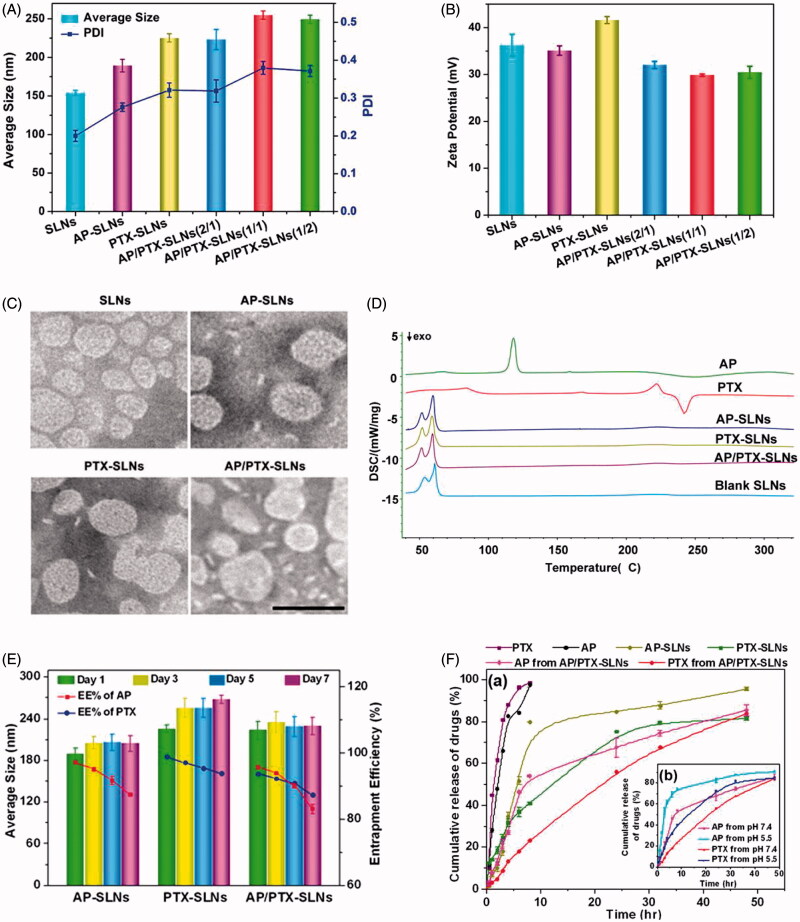
*In vitro* characterization of the AP/PTX-SLNs. (A) The average size and PDI of the SLNs, the AP-SLNs, the PTX-SLNs and the AP/PTX-SLNs. (B) The zeta potential of the four formulations. (C) TEM images of the four formulations. Scale bar: 200 nm. (D) Differential scanning calorimetry (DSC) thermograms of the AP/PTX-SLNs. (E) The encapsulation efficiency and stability of the AP-SLNs, the PTX-SLNs and the AP/PTX-SLNs at different time intervals. (F) Release profiles of AP and PTX from the formulations in PBS with 20% ethanol at pH 7.4 (a); the influence of pH (5.5 and 7.4) on the release of AP and PTX from the AP/PTX-SLNs (b).

Although major limitations of vitamin C for use as an anticancer drug can be solved by using more stable hydrophobized derivatives of ascorbate (such as ascorbyl palmitate), the poor water solubility and delivery efficiency of such derivatives remain deficient. To the best of our knowledge, nanoparticle delivery systems can improve the stability and solubility of drugs and improve drug pharmacokinetics. Therefore, in this study, a vitamin C derivative (ascorbyl palmitate, AP) and paclitaxel (PTX) dual-loaded composite solid lipid nanoparticles (SLNs) were developed to improve the antitumoral effects of AP and decrease the side effects of PTX. In detail, the dynamic light scattering (DLS) results showed that the average diameters of the blank SLNs, the AP-SLNs, the PTX-SLNs and the AP/PTX-SLNs with different AP/PTX ratios (2/1, 1/1 and 1/2) were 154, 189, 225, 223, 254 and 249 nm, respectively. These results suggested that the vitamin C derivative AP was successfully encapsulated into the SLNs with a size distribution of 223 nm, which was slightly larger than the blank SLNs (Figure 1(A)). The zeta potentials of the various SLNs were positively charged (Figure 1(B)) due to the positive DDAB that was used.

TEM images revealed that all SLNs formulations were spherical in shape, and their diameters were consistent with the nanoparticle size as measured by DLS (Figure 1(C)). Further characterization indicated that AP was encapsulated at extraordinarily high entrapment efficiencies of 97.66% in the AP-SLNs and 96.27% in the AP/PTX-SLNs. The entrapment efficiency of PTX was 98.8% in the PTX-SLNs and 93.58% in the AP/PTX-SLNs (Figure 1(E)). The drug-loading capacity of AP/PTX-SLNs was 6.3% for AP and 3.07% for PTX, respectively. It can be observed from Figure 1(E) that there were no significant changes in the mean particle size upon storing (*p* > .05). However, the encapsulation efficiencies of these formulations decreased with extended storage times, indicating that the AP/PTX-SLNs were not stable and required immediate preparation at the time of use or lyophilization for storage. Both AP and PTX were released more slowly from the AP/PTX-SLNs than from the single drug loaded SLNs (Figure 1(F)). AP and/or PTX exhibited sustained release from the AP-SLNs, PTX/SLNs and AP/PTX-SLNs for up to 48 h. In the initial 6 h, the released amount of AP reached 84.23%, whereas less than 25% was released from the AP/PTX-SLNs. Additionally, the drug release rates increased as the pH decreased from 7.4 to 5.5, which suggested that acidic conditions increased the release efficiency compared with neutral conditions. Generally, a pH of 5.5 simulates the tumor environment and the lysosomal environment. Therefore, enhanced AP/PTX release at pH 5.5 would aid in cancer therapies.

### 3.2. Differential scanning calorimetry (DSC) analysis

DSC was used to investigate the thermal properties of the nanoparticles, which can provide relevant physicochemical data concerning the state of the drug incorporated in the SLN formulations. Therefore, we examined the physical state of the drugs encapsulated in the SLNs via DSC. As shown in Figure 1(D), AP exhibited a melting endotherm peak at approximately 118 °C, and PTX revealed a melting peak at 221 °C and a decomposition peak at 242 °C, which implies that free AP and PTX are in the crystal state. After encapsulation inside the SLNs, all characteristic peaks of both drugs disappeared. It could be concluded that majority of AP and PTX formulated in the SLNs were in an amorphous or disordered crystalline phase molecular dispersion state in the lipid matrix (Xin et al., 2010). In addition, the polar part (ascorbate) of AP is hydrophilic, which can destabilize a hydrophobic matrix of SLNs immersed in the water. After the hydrophobic matrix was destroyed, the drugs in the amorphous state or disordered crystalline form could release from the lipid matrix more easily.

### 3.3. Cytotoxicity and synergistic effects of AP and PTX in the AP/PTX-SLNs

To verify the synergistic effect of this co-delivery system based on PTX and the vitamin C derivative (AP), the *in vitro* anticancer effects of the free drugs and the drug-loaded nanoparticles against B16F10 cells and A549 cells were evaluated via MTT assays. The cell viability histograms are shown in Figure S2. After 24 h of incubation, the cytotoxicity of AP, PTX, the AP + PTX, the AP-SLNs, the PTX-SLNs, the AP/PTX-SLNs and the AP-SLNs + PTX-SLNs on B16F10 cells was observed for different concentrations of AP and PTX, conforming to the dose-dependent cell proliferation inhibition behavior. The combination of AP and PTX led to enhanced cell proliferation inhibition. The AP/PTX-SLNs and the AP-SLNs + PTX-SLNs exhibited the best anticancer activity compared to AP, PTX, the AP + PTX, the AP-SLNs and the PTX-SLNs at AP concentrations from 2.5 to 20 μM (*p* < .05). When the concentration of AP increased to 40 μM, the cytotoxicity of the AP-SLNs, the PTX-SLNs and the AP/PTX-SLNs showed no significant differences in the cells (*p* > .05). The similar trend of cytotoxicity of AP/PTX-SLNs was observed in A549 cells (Figure S2B), suggesting that AP/PTX-SLNs were effective against different tumors. In AP-SLNs + PTX-SLNs group, both AP-SLNs and PTX-SLNs should be prepared separately and then subjected to combination use. These complex procedures make the co-loading of AP and PTX into the same SLN system valuable. To determine the optimal ratio of PTX to AP in the AP/PTX-SLNs for *in vitro* and *in vivo* treatment, the cytotoxicity of the AP/PTX-SLNs (with different mass ratios of 2/1, 1/1, and 1/2) against the B16F10 cell line was assessed. The IC_50_ values and combination index (CI_50_) values of the AP/PTX-SLNs were calculated and are summarized in Table S1. CI_50_ values lower than, equal to, or higher than ‘1’ indicate synergism, addition, or antagonism, respectively (Lv et al., 2014). According to the results, the CI_50_ value of the AP/PTX-SLNs (AP/PTX, 1/2) against the B16F10 cells was 1.38 ± 0.11, indicating no synergism against the B16F10 cells. The CI_50_ values of the AP/PTX-SLNs with AP/PTX ratios of 2/1 and 1/1 were 0.78 ± 0.10 and 1.01 ± 0.10, respectively. This result indicated that the AP/PTX-SLNs with an AP/PTX ratio of 2/1 (CI_50_=0.78) would provide the optimal synergistic combination of AP and PTX. Thus, this ratio was used for the following *in vitro* and *in vivo* experiments.

### 3.4. Cellular uptake of paclitaxel/vitamin C derivative composite SLNs loaded with paclitaxel

**Figure 2.**
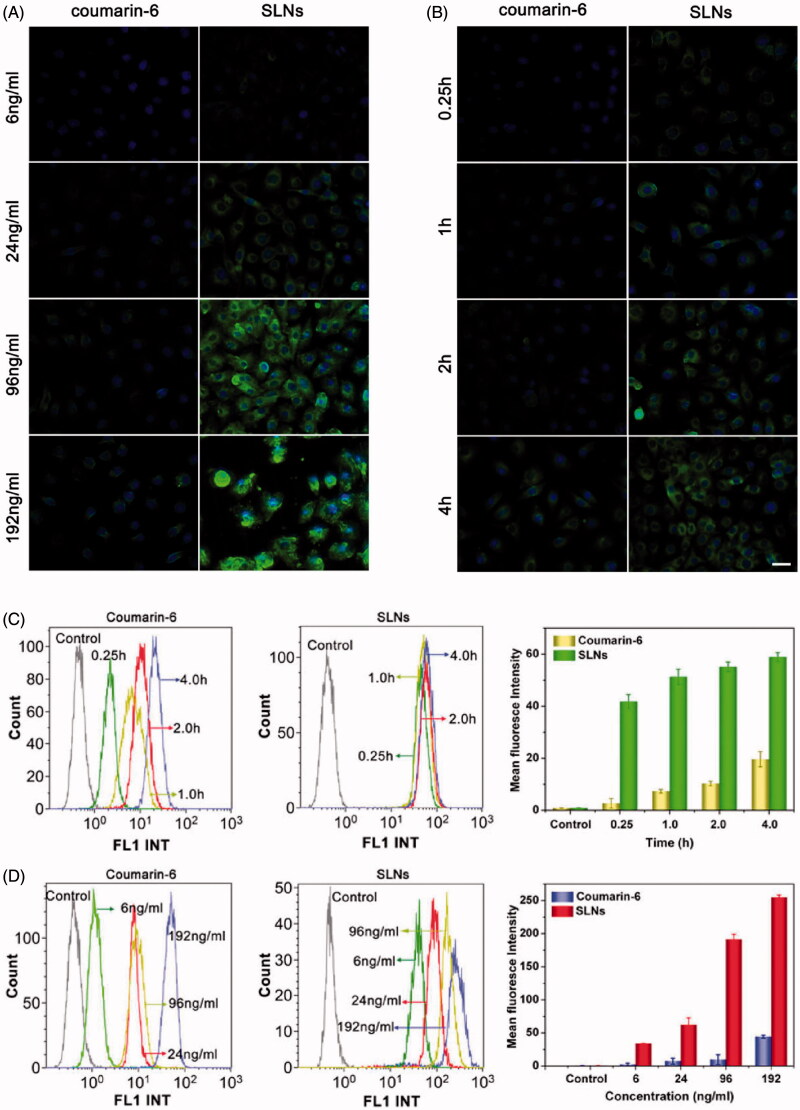
Cellular uptake of SLNs by the B16F10 cells illustrated by fluorescence images and flow cytometry histograms. Intracellular distribution of coumain-6-loaded SLNs for different coumain-6 concentrations (A) at different time points (B). The color green represents coumain-6-loaded SLNs absorbed by cells; blue is the color of cell nucleus stained with DAPI. Scale bar represents 40 μm. (C) The fluorescence intensity of the cells after incubation with free coumarin-6 and the SLNs at the indicated time points. (D) The fluorescence intensity of the cells after incubation with different concentrations of free coumarin-6 and the SLNs. The MFI of SLNs was larger than that of Coumarin-6 at corresponding time or concentration. The data are presented as the mean ± SD (*n* = 3).

The cellular uptake of the AP/PTX-SLNs was evaluated by fluorescence microscopy and flow cytometry. To facilitate detection of the nanoparticles in the cellular uptake studies, coumarin-6 was incorporated into the SLNs because coumarin-6 is widely used as a replacement fluorescent imaging marker of hydrophobic drugs (Zhang et al., 2010). In this study, the time- and concentration-dependent internalization of the SLNs was investigated. As exemplified in Figure 2, the B16F10 cells incubated with coumarin-6 labeled SLNs and free coumarin-6 demonstrated gradual increases in green fluorescence in the cytoplasm with time and with increasing concentration, indicating that the uptake of SLNs and free coumarin-6 were both time- and concentration-dependent. Compared with the free coumarin-6, the SLNs corresponded to significantly improved cellular uptake, which suggested that the SLNs could more effectively facilitate the uptake of drugs into cells. The mean fluorescence intensity was quantitatively assessed via flow cytometry (Figure 2(C,D)). Higher cellular uptake efficiencies were clearly observed for the SLNs groups compared with the coumarin-6, which further confirmed the result observed from the fluorescent microscopy (Figure 2). The qualitative and quantitative results showed that the SLNs exhibited increased cellular permeability and improved the cellular uptake of AP and PTX.

### 3.5. Cell apoptosis assay

**Figure 3.**
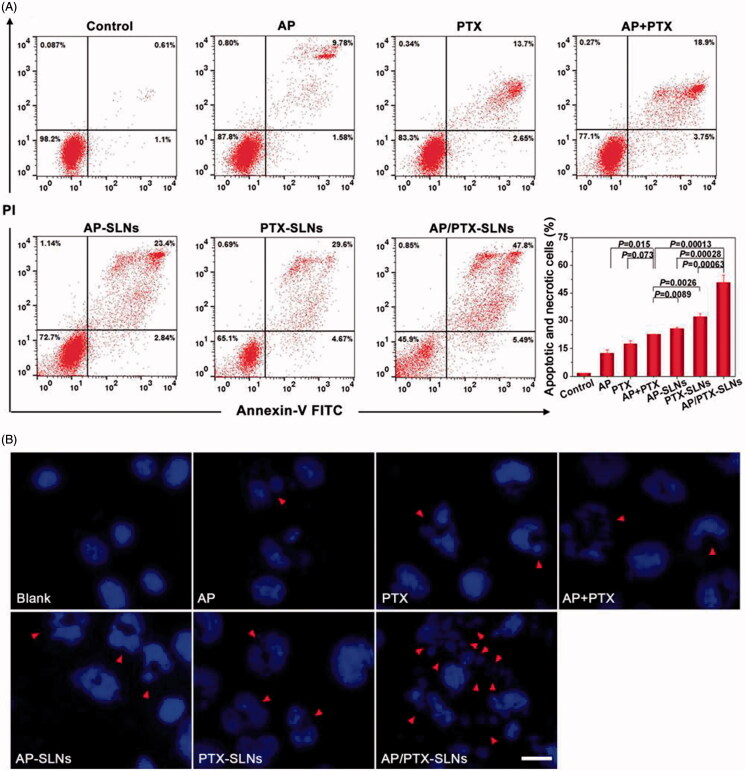
Antitumoral effects of the SLN formulations in the B16F10 cells *in vitro*. (A) Flow cytometric analysis of B16F10 cell apoptosis induced by the SLN formulations. (B) Cell apoptosis induction and morphological changes in the B16F10 cells induced by different formulations of AP and AP/PTX-SLNs (arrows: cell apoptosis). Scale bar represents 20 μm.

To investigate the influence of the AP/PTX-SLNs on cell apoptosis, we quantified the proportion of apoptotic cells by annexin V-FITC and PI double staining (Shen et al., 2012; Cao et al., 2015). As shown in Figure 3(A), AP/PTX-SLNs induced a significantly higher apoptotic and necrotic rate compared with other groups, including the B16F10 cells treated with the AP + PTX solution, the AP-SLNs and the PTX-SLNs (*p* < .05). The apoptotic and necrotic rate of the B16F10 cells treated with the free AP + PTX solution was extremely higher than that of the cells treated with the free AP and PTX solutions (*p* < .05), suggesting that the combined use of AP and PTX could increase the antitumoral efficiency.

The B16F10 cells were further stained by DAPI to evaluate the morphological changes of the cell nuclei by fluorescence microscopy. As shown in Figure 3(B), the nuclei in the B16F10 cells of the negative control group continued to exhibit a regular rounded shape with integrated homogeneous chromatin distributions after DAPI staining. In contrast, chromatin condensation, nuclear fragmentation and even apoptotic body production were observed in the free AP-, PTX-, AP + PTX-, AP-SLN-, PTX-SLN- and AP/PTX-SLN-treated groups. Among all formulations, the AP/PTX-SLNs induced the highest level of B16F10 cell apoptosis, suggesting a synergistic effect of the paclitaxel/vitamin C derivative composite nanoparticles.

### 3.6. Mechanism of the synergistic effect of AP/PTX-SLNs

**Figure 4.**
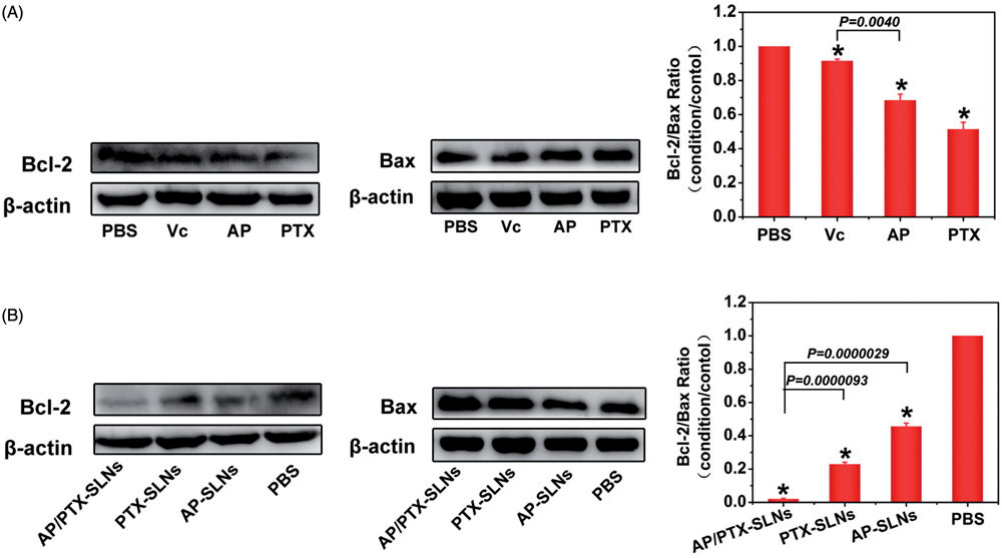
Drug treatment results in modulation of anti- and pro-apoptotic proteins (Bcl-2/Bax) in the B16F10 cells. (A) The cells were treated with Vc, AP, or PTX for 24 h. (B) The cells were treated with AP-SLNs, PTX-SLNs and AP/PTX-SLNs for 24 h. Densitometric measurements were assessed using LabQuant Software. The protein expression levels were normalized to those of PBS control (100%). The data are presented as the means ± SD (*n* = 3); *p < .05.

To further elucidate the observed enhanced anticancer effect of the composite SLN delivery system and to verify the synergistic effect imposed by paclitaxel and the vitamin C derivative, a combination index (CI_50_) assay and the potential molecular mechanism responsible for the co-delivery system were proposed. As observed in Table S1, the CI_50_ of AP/PTX-SLNs was 0.78 ± 0.10, which indicated a synergy in inhibiting cell proliferation. It has been reported that paclitaxel, a mitotic inhibitor, promotes tubulin polymerization and the formation of dysfunctional microtubules, disrupting the normal tubule dynamics required for cellular division and thus provoking cell death (Lv et al., 2014). The expression of Bcl-2, a typical apoptosis-related protein, potentiates the inhibition of tubulin polymerization that can be stimulated by Bax (Bcl-2 family) (Knipling & Wolff, 2006). Bcl-2, as the nodal point at the convergence of multiple pathways with broad relevance to oncology, is best known for its ability to suppress apoptosis (Yip & Reed, 2008). Decreased expression of Bcl-2 can induce apoptosis, while lack of Bax proteins protects cells from apoptosis (Cory & Adams, 2002). It has also been found that the ratio of Bcl-2 to Bax is important in deciding whether cells die or survive (Jun et al., 2007). Therefore, the decreasing of Bcl-2/Bax ratio in paclitaxel treatment is a sign of synergistic interaction. First, we investigated the expression of Bcl-2 and Bax in the B16F10 cells by western blotting after treatment with free Vc, AP and PTX for 24 h. As shown in Figure 4(A), there was a sight down-regulation of Bcl-2 expression in the PTX group, while the expression level of Bcl-2 in the Vc group was not significantly changed, probably because the dosage of Vc used in this antitumor study was too low. Additionally, the cellular uptake of Vc was limited by its intrinsic water solubility property. Compared with the Vc group, the Bax expression level in the PTX or AP group was up-regulated and the Bcl-2/Bax ratio was decreased in the following order from greatest to lowest: Vc > AP > PTX. However, increasing the liposolubility of Vc in the form of AP can improve the efficacy of down-regulating Bcl-2 expression and up-regulating Bax expression. These results conformed to the conclusion that only high doses of ascorbic acid have the ability to induce cancer cell death. Therefore, a simple and valid drug delivery system is highly desired for improving the drug delivery efficacy against tumor cells. In light of this, SLN-based delivery is one approach that can be used to solve this problem.

After precisely loading drugs into the SLNs, we investigated the expression of Bcl-2 and Bax after treatment with the AP-SLNs, the PTX-SLNs and the AP/PTX-SLNs. As shown in Figure 4(B), the levels of Bcl-2 in the B16F10 cells were reduced upon treatment for 24 h with the various SLNs formulations. The AP/PTX-SLNs almost completely depleted the expression of Bcl-2 and were more effective than other groups. On the contrary, the expression levels of Bax were increased significantly in the AP/PTX-SLN group. Compared with PTX- or AP-loaded SLNs, AP/PTX-SLNs showed the much lower Bcl-2/Bax ratio in B16F10 cells (*p* < .05). Conclusively, the AP/PTX-SLNs exhibited a greater efficacy in inducing cell apoptosis by reducing the Bcl-2/Bax ratio accompanied by promoting tubulin polymerization (Knipling & Wolff, 2006).

### 3.7. *In vivo* imaging for biodistribution

**Figure 5.**
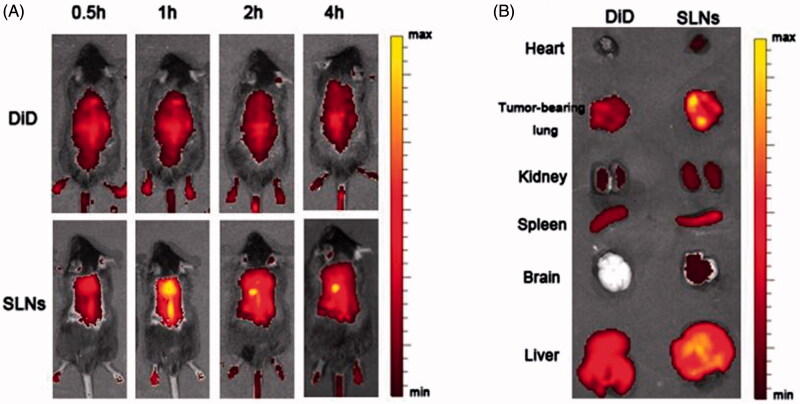
*In vivo* fluorescence images of SLNs in mice and mature SLN levels in the tumor-bearing lungs. (A) Time-dependent intensity images of the fluorescence distribution in the mice. (B) *In vivo* fluorescence images of major organs at 2 h after injection of SLNs.

Fluorescence imaging was employed to monitor the *in vivo* biodistribution and tumor targeting characteristics of the SLNs containing DDAB. DiD fluorescence probe and DiD-labeled SLNs were administered intravenously into the mice though the tail vein, and DiD signals were measured at pre-determined time points using an IVIS^®^ Spectrum system to capture the distribution patterns in live mice. As shown in Figure 5(A), fluorescence signals could be observed in the tumor-bearing lungs as early as 0.5 h after injection and exhibited a maximum fluorescence signal at 1.0 h post-injection that decreased after 2 h post-injection. Compared with the SLN group, the fluorescence signals of the free DiD group were much weaker at all time points post-injection and were primarily located in the liver. After 2 h, the mice were sacrificed, and the major organs from the euthanized animals were also imaged under the same set of parameters. As expected in the SLNs group, a dramatic increase in the DiD signal was observed in the tumor-bearing lungs, which indicates that many SLNs accumulated in the tumor-bearing lungs, primarily due to the ability of the delivery vehicle to be physically entrapped in the capillary bed of the lungs. Thus, it serves as a drug reservoir that can facilitate such nanocarriers to permeate into tumor sites because of the enhanced permeation and retention (EPR) effect.

### 3.8. *In vivo* anti-cancer efficacy of the AP/PTX-SLNs in the B16F10 lung metastasis tumor model

**Figure 6.**
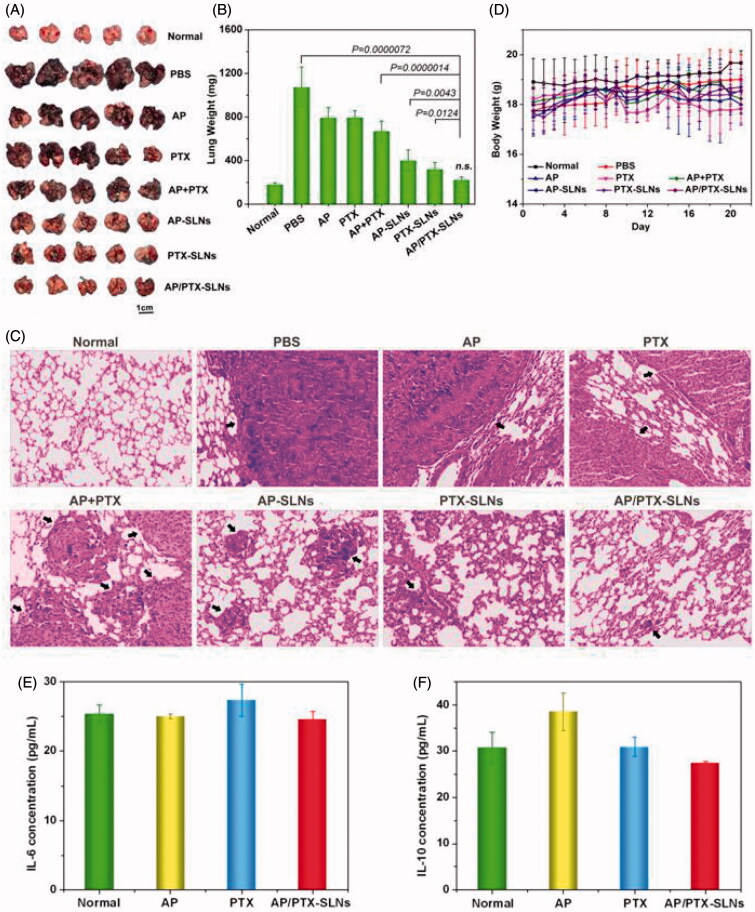
The improved antitumor efficiency and preliminary safety evaluation of the AP/PTX-SLNs. (A) Images of the B16F10-bearing lungs on day 21 after seven consecutive i.v. injections of AP/PTX-SLNs (*n* = 5). (B) The antitumoral effects of different treatments evaluated by the B16F10-bearing lung weight (*n* = 5); n.s., not significant. (C) Histological staining of the B16F10-bearing lungs after various treatments (arrows: tumor nodules). (D) Body weight monitoring of the B16F10-bearing mice after different treatments (*n* = 5). Serum concentrations of IL-6 (E) and IL-10 (F) in the C57BL/6 mice at 6 h after intravenous injection with the AP/PTX-SLNs.

In this study, *in situ* lung metastasis tumor-bearing mice were utilized to assess the effect of the AP/PTX-SLNs on tumor inhibition. Their tumor morphology and weights were monitored, and histological analysis and TUNEL staining analyses were also performed. As shown in Figure 6(A), the lungs excised from the normal group exhibited normal physiological morphology. However, tumor nodules covered nearly the entire surface of the lungs in the untreated tumor-bearing mice (PBS group). The B16F10-bearing tumor nodules that were detected in the lungs of the AP-, PTX- and AP + PTX-treated groups were decreased in the mice that were treated with the drug-loaded SLNs (AP-SLNs, PTX-SLNs and AP/PTX-SLNs), especially in the AP/PTX-SLN-treated group. The average weights of the tumor-bearing lung tissues were calculated and are shown in Figure 6(B). As expected, significant decreases in the B16F10-bearing lung weights were found in all drug-containing SLN groups compared with the PBS and free drug groups (*p* < .05). More importantly, the AP/PTX-SLNs exhibited the highest antitumoral efficiency compared with the free drug groups (AP, PTX and AP + PTX) and single drug-loaded SLN (AP-SLNs and PTX-SLNs) groups (*p* < .05). The average body weight of the AP/PTX-SLNs groups was not significantly different compared to the normal groups (*p* > .05). Furthermore, the histopathology of the tumor-bearing lung tissues was further analyzed by observing the hematoxylin and eosin (H&E)-stained slides under an optical microscope. As shown in Figure 6(C), most of the lung tissues treated with the AP/PTX-SLNs contained noticeably lower densities of tumor cells than those of the other groups (AP, PTX, AP + PTX, AP-SLNs and PTX-SLNs) (Figure 6(C)). Moreover, the tumor-bearing mice treated with the AP/PTX-SLNs exhibited less tumor lesions and more healthy alveoli than the single drug-treated mice, which was consistent with the macroscopic observations. The *in vivo* experiments supported the synergism of AP and PTX in the SLNs. It can also be observed from Figure 6(D) that body weights of the mice did not decrease following treatment, indicating that no significant toxicity occurred in these groups within the experimental periods. The tumor-bearing lung tissues were further sectioned after treatment for TUNEL staining. The results showed a similar trend of cell apoptosis induced by the formulations (Figure S3). The SLNs loaded with dual drugs induced the highest levels of cell apoptosis among the various formulations (Figure S3), further validating the combined administration effect of AP and PTX on B16F10 tumors. The free drugs induced few apoptotic cells *in vivo*, probably owing to the low level of drug concentration in the tumor sites mediated by the EPR effect. Moreover, free drugs in the serum were metabolized and cleared more quickly than drugs bounded in the nanoparticles before accumulating in the tumor. Therefore, it can be concluded from these findings that SLNs improve the accumulation of drugs in the tumor and promote antitumoral efficiency. In addition, vitamin C, including AP, could facilitate paclitaxel chemotherapy *in vivo*, and a simple and valid delivery system is essential for improving the efficacy of chemotherapy. The inhibition efficacy of the formulations decreased in the following order from most effective to lease effective: AP/PTX-SLNs > PTX-SLNs > AP-SLNs > AP + PTX > PTX > AP.

### 3.9. Preliminary toxicity evaluation

In addition to characterization of the anticancer efficacy of the AP/PTX-SLNs *in vivo*, we also evaluated their *in vivo* toxicity and inflammation in terms of histological examinations and serum concentrations of IL-6 and IL-10. Healthy C57BL/6 mice were i.v. injected with different drug formulations at a PTX dose of 4 mg/kg, which was equal to the therapeutic dose. Histopathology confirmed the absence of inflammatory responses in the tissue following administration of the AP/PTX-SLNs (Figure S4), which suggests that the SLN formulations are biocompatible.

Other hematological parameters such as IL-6 and IL-10 were also examined to investigate whether the AP/PTX-SLNs induced an immune response, and the results are shown in Figure 6(E, F). Compared to untreated control mice, both IL-6 and IL-10 were within the normal levels (*p* > .05). Therefore, the AP/PTX synergistic combination-based SLN therapy did not induce toxicity and represents a promising strategy for paclitaxel/the vitamin C derivative in promoting anti-cancer effects.

## 4. Conclusions

In summary, we exploited a drug delivery system carrying ascorbyl palmitate and paclitaxel for synergistic/improved anticancer therapy. The obtained AP/PTX-loaded solid lipid nanoparticles were spherical with an average size of 223 nm, as observed by dynamic light scattering. These systems not only exhibited high levels of encapsulation efficiency (96.27% for AP, 93.58% for PTX) but also increased cellular permeability and improved the cellular uptake of drugs in B16F10 cells. The *in vitro* cytotoxicity test showed that the AP/PTX-SLNs with an AP/PTX ratio of 2/1 provided the optimal synergistic anticancer efficacy of AP and PTX. The cationic SLNs facilitated the accumulation of drugs into the lungs and could be used as drug carriers to treat lung cancer with low toxicity. Overall, this paclitaxel/vitamin C derivative composite nanoparticle system enhanced the therapeutic tumor suppression of PTX via a synergistic effect that reduces the Bcl-2/Bax ratio accompanied by promoting tubulin polymerization.

## References

[CIT0001] Austria R, Semenzato A, Bettero A. (1997). Stability of vitamin C derivatives in solution and topical formulations. J Pharm Biomed Anal 15:795–801. 9172105 10.1016/s0731-7085(96)01904-8

[CIT0002] Banks WA, Kastin AJ. (1985). Peptides and the blood-brain barrier: lipophilicity as a predictor of permeability. Brain Res Bull 15:287–92. 2413968 10.1016/0361-9230(85)90153-4

[CIT0003] Cameron E, Campbell A. (1974). The orthomolecular treatment of cancer. II. Clinical trial of high-dose ascorbic acid supplements in advanced human cancer. Chemico-Biol Interact 9:285–315. 10.1016/0009-2797(74)90019-2 4430016

[CIT0004] Cameron E, Pauling L. (1976). Supplemental ascorbate in the supportive treatment of cancer: prolongation of survival times in terminal human cancer. Proc Natl Acad Sci USA 73:3685–9. 1068480 10.1073/pnas.73.10.3685 PMC431183

[CIT0005] Cao X, Luo J, Gong T, et al. (2015). Coencapsulated doxorubicin and bromotetrandrine lipid nanoemulsions in reversing multidrug resistance in breast cancer in vitro and in vivo. Mol Pharm 12:274–86. 25469833 10.1021/mp500637b

[CIT0006] Chen Q, Espey MG, Sun AY, et al. (2008). Pharmacologic doses of ascorbate act as a prooxidant and decrease growth of aggressive tumor xenografts in mice. Proc Natl Acad Sci USA 105:11105–9. 18678913 10.1073/pnas.0804226105 PMC2516281

[CIT0007] Chen Y, Zhu X, Zhang X, et al. (2010). Nanoparticles modified with tumor-targeting scFv deliver siRNA and miRNA for cancer therapy. Mol Ther 18:1650–6. 20606648 10.1038/mt.2010.136 PMC2956922

[CIT0008] Cory S, Adams JM. (2002). The Bcl2 family: regulators of the cellular life-or-death switch. Nat Rev Cancer 2:647–56. 12209154 10.1038/nrc883

[CIT0009] Creagan ET, Moertel CG, O’Fallon JR, et al. (1979). Failure of high-dose vitamin C (ascorbic acid) therapy to benefit patients with advanced cancer. A controlled trial. The. N Engl J Med 301:687–90. 384241 10.1056/NEJM197909273011303

[CIT0010] D’Souza GG, Wang T, Rockwell K, Torchilin VP. (2008). Surface modification of pharmaceutical nanocarriers with ascorbate residues improves their tumor-cell association and killing and the cytotoxic action of encapsulated paclitaxel in vitro. Pharm Res 25:2567–72. 18618230 10.1007/s11095-008-9674-4

[CIT0011] Du J, Martin SM, Levine M, et al. (2010). Mechanisms of ascorbate-induced cytotoxicity in pancreatic cancer. Clin Cancer Res 16:509–20. 20068072 10.1158/1078-0432.CCR-09-1713 PMC2807999

[CIT0012] Espey MG, Chen P, Chalmers B, et al. (2011). Pharmacologic ascorbate synergizes with gemcitabine in preclinical models of pancreatic cancer. Free Radical Biol Med 50:1610–9. 21402145 10.1016/j.freeradbiomed.2011.03.007 PMC3482496

[CIT0013] Guney G, Kutlu HM, Genc L. (2014). Preparation and characterization of ascorbic acid loaded solid lipid nanoparticles and investigation of their apoptotic effects. Colloids Surf B Biointerfaces 121:270–80. 24985762 10.1016/j.colsurfb.2014.05.008

[CIT0014] Guo D, Dou D, Li X, et al. (2017). Ivermection-loaded solid lipid nanoparticles: preparation, characterisation, stability and transdermal behaviour. Artificial Cells Nanomed Biotechnol. DOI: 10.1080/21691401.2017.1307207. 28368657

[CIT0015] Ha YM, Park MK, Kim HJ, et al. (2009). High concentrations of ascorbic acid induces apoptosis of human gastric cancer cell by p38-MAP kinase-dependent up-regulation of transferrin receptor. Cancer Lett 277:48–54. 19108947 10.1016/j.canlet.2008.11.020

[CIT0016] Hardaway CM, Badisa RB, Soliman KF. (2012). Effect of ascorbic acid and hydrogen peroxide on mouse neuroblastoma cells. Mol Med Rep 5:1449–52. 22469841 10.3892/mmr.2012.857 PMC3327822

[CIT0017] He C, Liu D, Lin W. (2015). Self-assembled nanoscale coordination polymers carrying siRNAs and cisplatin for effective treatment of resistant ovarian cancer. Biomaterials 36:124–33. 25315138 10.1016/j.biomaterials.2014.09.017 PMC4252980

[CIT0018] Jun HS, Park T, Lee CK, et al. (2007). Capsaicin induced apoptosis of B16-F10 melanoma cells through down-regulation of Bcl-2. Food Chem Toxicol 45:708–15. 17306913 10.1016/j.fct.2006.10.011

[CIT0019] Kang JS, Cho D, Kim YI, et al. (2003). L-ascorbic acid (vitamin C) induces the apoptosis of B16 murine melanoma cells via a caspase-8-independent pathway. Cancer Immunol Immunother 52:693–8. 12827307 10.1007/s00262-003-0407-6 PMC11032859

[CIT0020] Knipling L, Wolff J. (2006). Direct interaction of Bcl-2 proteins with tubulin. Biochem Biophys Res Commun 341:433–9. 16446153 10.1016/j.bbrc.2005.12.201

[CIT0021] Kristl J, Volk B, Gasperlin M, Sentjurc M, Jurkovic P. (2003). Effect of colloidal carriers on ascorbyl palmitate stability. Eur J Pharm Sci 19:181–9. 12885382 10.1016/s0928-0987(03)00104-0

[CIT0022] Lee KM, Kwon JY, Lee KW, Lee HJ. (2009). Ascorbic acid 6-palmitate suppresses gap-junctional intercellular communication through phosphorylation of connexin 43 via activation of the MEK-ERK pathway. Mutat Res 660:51–6. 19026667 10.1016/j.mrfmmm.2008.10.012

[CIT0023] Lin ZY, Chuang WL. (2010). Pharmacologic concentrations of ascorbic acid cause diverse influence on differential expressions of angiogenic chemokine genes in different hepatocellular carcinoma cell lines. Biomed Pharmacother 64:348–51. 19932582 10.1016/j.biopha.2009.06.005

[CIT0024] Lv S, Tang Z, Li M, et al. (2014). Co-delivery of doxorubicin and paclitaxel by PEG-polypeptide nanovehicle for the treatment of non-small cell lung cancer. Biomaterials 35:6118–29. 24794923 10.1016/j.biomaterials.2014.04.034

[CIT0025] Maeda H, Nakamura H, Fang J. (2013). The EPR effect for macromolecular drug delivery to solid tumors: Improvement of tumor uptake, lowering of systemic toxicity, and distinct tumor imaging in vivo. Adv Drug Deliv Rev 65:71–9. 23088862 10.1016/j.addr.2012.10.002

[CIT0026] Mc CW. (1959). Cancer: a collagen disease, secondary to a nutritional deficiency. Arch Pediatr 76:166–71. 13638066

[CIT0027] Miles SL, Fischer AP, Joshi SJ, et al. (2015). Ascorbic acid and ascorbate-2-phosphate decrease HIF activity and malignant properties of human melanoma cells. BMC Cancer 15:867. 26547841 10.1186/s12885-015-1878-5 PMC4636772

[CIT0028] Miwa N, Yamazaki H, Nagaoka Y, et al. (1988). Altered production of the active oxygen species is involved in enhanced cytotoxic action of acylated derivatives of ascorbate to tumor cells. Biochimica Et Biophysica Acta 972:144–51. 3191161 10.1016/0167-4889(88)90113-9

[CIT0029] Moertel CG, Fleming TR, Creagan ET, et al. (1985). High-dose vitamin C versus placebo in the treatment of patients with advanced cancer who have had no prior chemotherapy. A randomized double-blind comparison. N Engl J Med 312:137–41. 3880867 10.1056/NEJM198501173120301

[CIT0030] Muller RH, Mader K, Gohla S. (2000). Solid lipid nanoparticles (SLN) for controlled drug delivery - a review of the state of the art. Eur J Pharm Biopharm 50:161–77. 10840199 10.1016/s0939-6411(00)00087-4

[CIT0031] Naidu KA. 2003a. Ascorbyl esters for the treatment of cancer. United States Patent. 6638974.

[CIT0032] Naidu KA. (2003b). Vitamin C in human health and disease is still a mystery? An overview. Nutr J 2:7. 14498993 10.1186/1475-2891-2-7 PMC201008

[CIT0033] Ohno S, Ohno Y, Suzuki N, et al. (2009). High-dose vitamin C (ascorbic acid) therapy in the treatment of patients with advanced cancer. Anticancer Res 29:809–15. 19414313

[CIT0034] Padayatty SJ, Sun H, Wang Y, et al. (2004). Vitamin C pharmacokinetics: implications for oral and intravenous use. Ann Intern Med 140:533–7. 15068981 10.7326/0003-4819-140-7-200404060-00010

[CIT0035] Pathi SS, Lei P, Sreevalsan S, et al. (2011). Pharmacologic doses of ascorbic acid repress specificity protein (Sp) transcription factors and Sp-regulated genes in colon cancer cells. Nutr Cancer 63:1133–42. 21919647 10.1080/01635581.2011.605984 PMC3359146

[CIT0036] Perrin C, Meyer L. (2003). Simultaneous determination of ascorbyl palmitate and nine phenolic antioxidants in vegetable oils and edible fats by HPLC. J Amer Oil Chem Soc 80:115–8.

[CIT0037] Pollard HB, Levine MA, Eidelman O, Pollard M. (2010). Pharmacological ascorbic acid suppresses syngeneic tumor growth and metastases in hormone-refractory prostate cancer. In Vivo 24:249–55. 20554995 PMC9718485

[CIT0038] Qu J, Zhang L, Chen Z, et al. (2016). Nanostructured lipid carriers, solid lipid nanoparticles, and polymeric nanoparticles: which kind of drug delivery system is better for glioblastoma chemotherapy? Drug Deliv 23:3408–16. 27181462 10.1080/10717544.2016.1189465

[CIT0039] Sawant RR, Vaze O, D’Souza GG, et al. (2011). Palmitoyl ascorbate-loaded polymeric micelles: cancer cell targeting and cytotoxicity. Pharm Res 28:301–8. 20730558 10.1007/s11095-010-0242-3

[CIT0040] Shen J, Yin Q, Chen L, et al. (2012). Co-delivery of paclitaxel and survivin shRNA by pluronic P85-PEI/TPGS complex nanoparticles to overcome drug resistance in lung cancer. Biomaterials 33:8613–24. 22910221 10.1016/j.biomaterials.2012.08.007

[CIT0041] Shi F, Zhao JH, Liu Y, et al. (2012). Preparation and characterization of solid lipid nanoparticles loaded with frankincense and myrrh oil. Int J Nanomed 7:2033–43. 10.2147/IJN.S30085 PMC3356207 22619540

[CIT0042] Silva AC, Gonzalez-Mira E, Garcia ML, et al. (2011). Preparation, characterization and biocompatibility studies on risperidone-loaded solid lipid nanoparticles (SLN): high pressure homogenization versus ultrasound. Colloids Surf B Biointerfaces 86:158–65. 21530187 10.1016/j.colsurfb.2011.03.035

[CIT0043] Takemura Y, Satoh M, Satoh K, et al. (2010). High dose of ascorbic acid induces cell death in mesothelioma cells. Biochem Biophys Res Commun 394:249–53. 20171954 10.1016/j.bbrc.2010.02.012

[CIT0044] Verrax J, Calderon PB. (2009). Pharmacologic concentrations of ascorbate are achieved by parenteral administration and exhibit antitumoral effects. Free Radical Biol Med 47:32–40. 19254759 10.1016/j.freeradbiomed.2009.02.016

[CIT0045] Wang B, Yu XC, Xu SF, et al. (2015a). Paclitaxel and etoposide co-loaded polymeric nanoparticles for the effective combination therapy against human osteosarcoma. J Nanobiotechnol 13:22. 10.1186/s12951-015-0086-4 PMC4377179 25880868

[CIT0046] Wang H, Zhao Y, Wu Y, et al. (2011). Enhanced anti-tumor efficacy by co-delivery of doxorubicin and paclitaxel with amphiphilic methoxy PEG-PLGA copolymer nanoparticles. Biomaterials 32:8281–90. 21807411 10.1016/j.biomaterials.2011.07.032

[CIT0047] Wang J, Wang H, Zhu R, et al. (2015b). Anti-inflammatory activity of curcumin-loaded solid lipid nanoparticles in IL-1beta transgenic mice subjected to the lipopolysaccharide-induced sepsis. Biomaterials 53:475–83. 25890744 10.1016/j.biomaterials.2015.02.116

[CIT0048] Wang W, Xi M, Duan X, et al. (2015c). Delivery of baicalein and paclitaxel using self-assembled nanoparticles: synergistic antitumor effect in vitro and in vivo. Int J Nanomed 10:3737–50. 10.2147/IJN.S80297 PMC4447173 26045664

[CIT0049] Xin H, Chen L, Gu J, et al. (2010). Enhanced anti-glioblastoma efficacy by PTX-loaded PEGylated poly(varepsilon-caprolactone) nanoparticles: in vitro and in vivo evaluation. Int J Pharm 402:238–47. 20934500 10.1016/j.ijpharm.2010.10.005

[CIT0050] Yip KW, Reed JC. (2008). Bcl-2 family proteins and cancer. Oncogene 27:6398–406. 18955968 10.1038/onc.2008.307

[CIT0051] Yoksan R, Jirawutthiwongchai J, Arpo K. (2010). Encapsulation of ascorbyl palmitate in chitosan nanoparticles by oil-in-water emulsion and ionic gelation processes. Colloids Surf B Biointerfaces 76:292–7. 20004558 10.1016/j.colsurfb.2009.11.007

[CIT0052] Zhang P, Hu L, Wang Y, et al. (2010). Poly(epsilon-caprolactone)-block-poly(ethyl ethylene phosphate) micelles for brain-targeting drug delivery: in vitro and in vivo valuation. Pharm Res 27:2657–69. 20848303 10.1007/s11095-010-0265-9

